# Cloud-scale RNA-sequencing differential expression analysis with Myrna

**DOI:** 10.1186/gb-2010-11-8-r83

**Published:** 2010-08-11

**Authors:** Ben Langmead, Kasper D Hansen, Jeffrey T Leek

**Affiliations:** 1 Department of Biostatistics, Johns Hopkins Bloomberg School of Public Health, 615 North Wolfe Street, Baltimore, MD 21205, USA

## Abstract

As sequencing throughput approaches dozens of gigabases per day, there is a growing need for efficient software for analysis of transcriptome sequencing (RNA-Seq) data. Myrna is a cloud-computing pipeline for calculating differential gene expression in large RNA-Seq datasets. We apply Myrna to the analysis of publicly available data sets and assess the goodness of fit of standard statistical models. Myrna is available from http://bowtie-bio.sf.net/myrna.

## Rationale

As cost and throughput continue to improve, second generation sequencing [1], in conjunction with RNA-Seq [2,3], is becoming an increasingly efficient and popular tool for studying gene expression. Currently, an RNA-Seq sequencing run generates hundreds of millions of reads derived from coding mRNA molecules in one or more biological samples. A typical RNA-Seq differential-expression analysis proceeds in three stages. First, reads are computationally categorized according to the transcribed feature from which each likely originated. Features of interest could be genes, exons or isoforms. This categorization might be conducted comparatively with respect to a reference [4], by *de novo *assembly [5], or a combination of both [6-8]. Second, a normalized count of the number of reads assigned to each feature is calculated. The count acts as a proxy for the feature's true abundance in the sample. Third, a statistical test is applied to identify which features exhibit differential abundance, or expression, between samples.

Since second generation sequencing produces a very large number of reads distributed across the entire transcriptome, RNA-Seq affords greater resolution than expression arrays. Preliminary comparisons of the data from RNA-Seq also suggest that the measurements may more precisely measure RNA abundance in spike-in experiments than gene expression microarrays, provided appropriate normalization is applied [4,9].

But improvements in sequencing cost and throughput also pose a data analysis challenge. While sequencing throughput grows at a rate of about 5× per year [10-12], computer speeds are thought to double approximately every 18 or 24 months [13]. Recent studies and commentaries [13-17] propose cloud computing as a paradigm that counteracts this disparity by tapping into the economies of scale afforded by commercial and institutional computing centers. If an algorithm can be made to run efficiently on many loosely coupled processors, implementing it as a cloud application makes it particularly easy to exploit the resources offered by large utility-computing services. These include commercial services such as Amazon's Elastic Compute Cloud [18] and Elastic MapReduce [19] services, or non-commercial services such as the IBM/Google Cloud Computing University Initiative [20] and the US Department of Energy's Magellan service [21].

**Figure 1:**
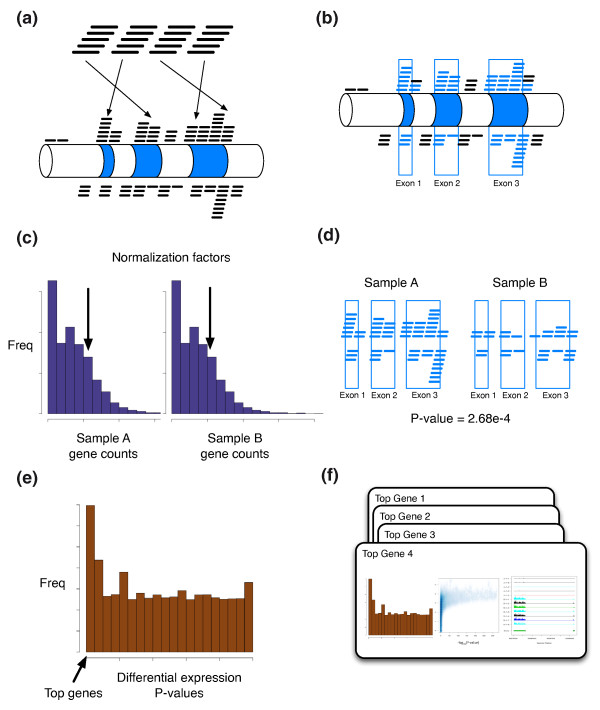
**The Myrna pipeline**. **(a) **Reads are aligned to the genome using a parallel version of Bowtie. **(b) **Reads are aggregated into counts for each genomic feature - for example, for each gene in the annotation files. **(c) **For each sample a normalization constant is calculated based on a summary of the count distribution. **(d) **Statistical models are used to calculate differential expression in the R programming language parallelized across multiple processors. **(e) **Significance summaries such as *P*-values and gene-specific counts are calculated and returned. **(f) **Myrna also returns publication ready coverage plots for differentially expressed genes.

Here we present Myrna, a cloud computing tool for calculating differential gene expression in large RNA-Seq datasets. Myrna integrates short read alignment with interval calculations, normalization, aggregation and statistical modeling in a single computational pipeline. After alignment, Myrna calculates coverage for exons, genes, or coding regions and differential expression using either parametric or non-parametric permutation tests. The results are returned in the form of per-gene *P*-values and *Q*-values for differential expression, a raw count table, an RPKM table (of reads per kilobase of exon model per million mapped reads), coverage plots for significant genes that can be directly incorporated into publications (Figure 1), and other diagnostic plots.

We apply Myrna to the analysis of a large publicly available RNA-Seq data set. One major advantage of our cloud-based implementation is the ability to rapidly test multiple plausible models for RNA-Seq differential expression. It has been suggested that this type of flexibility is necessary for computational applications to keep pace with the rapidly increasing number of reads in next-generation sequencing data sets [13]. Using Myrna we show that biological replicates reflect substantially increased variation compared to technical replicates in RNA-Seq and demonstrate that the commonly used Poisson model is not appropriate for biological replicates.

Myrna is designed with a parallel Hadoop/MapReduce model in mind. Myrna can be run on the cloud using Amazon Elastic MapReduce, on any Hadoop cluster, or on a single computer (without requiring Hadoop).

## Results

### Analysis of HapMap expression data

We applied Myrna to the analysis of a large population-based RNA-Seq experiment [22]. This experiment sequenced 69 lymphoblastoid cell lines derived from unrelated Nigerian individuals studied by the HapMap project [23], the largest publicly available RNA-Seq experiment at the time of writing. Each sample was sequenced at two separate labs (Argonne and Yale) on Illumina Genome Analyzer II instruments. For each sample, both labs contributed at least one lane of unpaired reads. In cases where a lab contributed more than one lane, we excluded data from all lanes beyond the first. The total input consisted of 1.1 billion reads; one center generated 35-bp unpaired reads and the other 46-bp unpaired reads. All reads were truncated to 35 bp prior to alignment. For each gene, a minimal set of genomic intervals was calculated such that all bases covered by the interval set were covered by all annotated gene transcripts. Where intervals for two or more genes overlapped, the overlapping subinterval was excluded from all sets. The result is one non-overlapping interval set per gene encoding the portions of the gene that are 'constitutive' (included in all transcripts) according to the annotation, and unique to that gene. Reads were aligned with Bowtie [24] using quality scores and requiring that only reads with a single best alignment are retained. Instances where the base at the extreme 3' end of a read aligned inside a gene's minimal interval set were calculated, each such instance counting as an 'overlap' between the gene and the sample from which the read originated. For this experiment, about 594 million reads (54%) aligned uniquely, whereas about 412 million (38%) aligned non-uniquely and were discarded, and about 97 million (8.8%) failed to align. Of the 594 million reads that aligned uniquely, about 189 million (32% of the reads that aligned uniquely, 17.1% of the input reads) overlapped the minimal interval set for a gene.

For our analysis, we pooled all reads from both labs for each sample. After pooling, Myrna filtered all genes without any counts, resulting in 14,934 genes with between 1 and 5,087,304 counts.

We used Myrna to analyze the HapMap data using six different statistical models for significance. The first pair of models used a test statistic based on a Poisson distribution, the second pair used a test statistic based on a Gaussian distribution (the well known *t*-test) for the log-transformed counts, and the third pair calculated statistics using the same Gaussian based test statistic, but used a permutation approach to calculate significance (see Materials and methods). For each of these distributional assumptions we performed one of two types of normalization: 75th percentile normalization [4] or a new normalization procedure where the 75th percentile is included as a term in the statistical model (see Materials and methods). We applied these methods to the HapMap data after randomly assigning each sample to one of two groups. In this case, we expect no differential expression signal, and *P*-values from these tests should be uniformly distributed.

**Figure 2:**
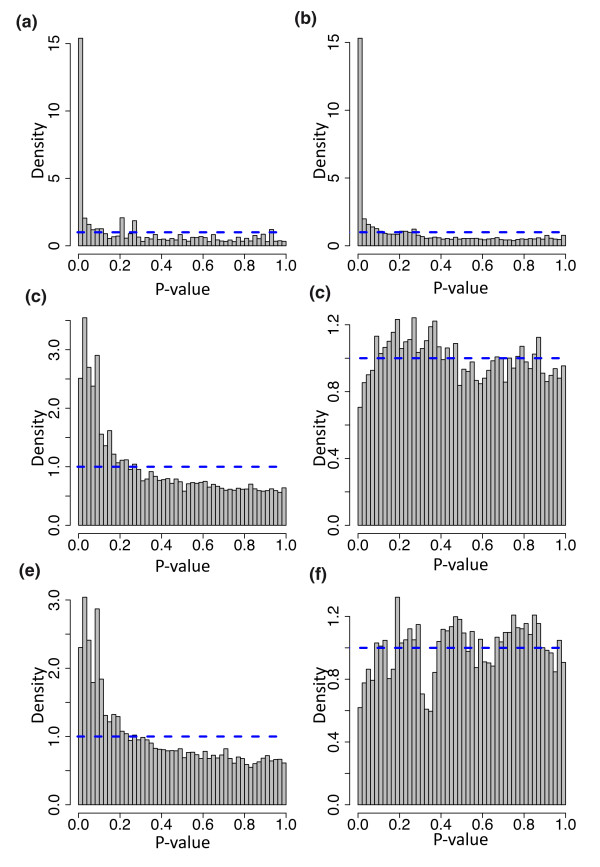
**Hapmap results**. Histograms of *P*-values from six different analysis strategies applied to randomly labeled samples. In each case the *P*-values should be uniformly distributed (blue dotted line) since the labels are randomly assigned. **(a) **Poisson model, 75th percentile normalization. **(b) **Poisson model, 75th percentile included as term. **(c) **Gaussian model, 75th percentile normalization. **(d) **Gaussian model, 75th percentile included as term. **(e) **Permutation model, 75th percentile normalization. **(f) **Permutation model, 75th percentile included as term.

**Figure 3:**
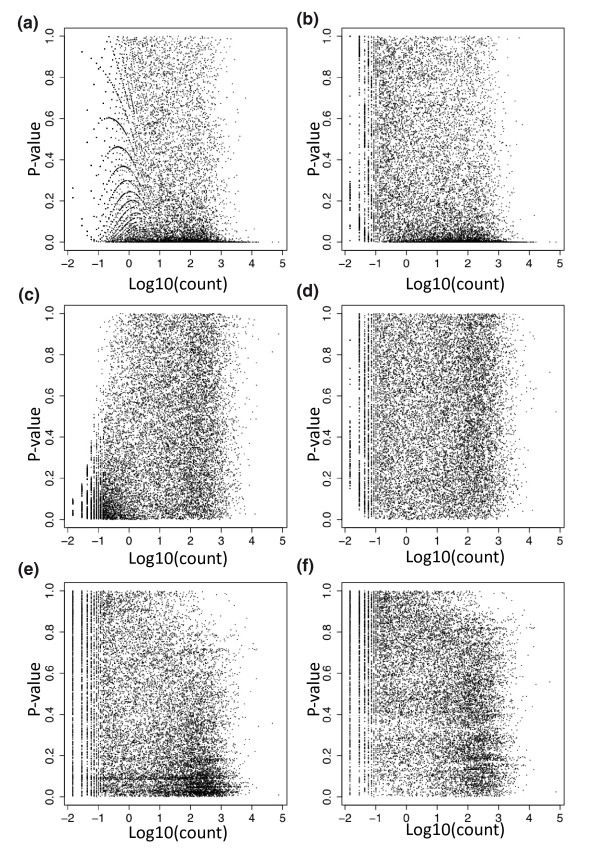
**Hapmap *P*-values versus read depth**. A plot of *P*-value versus the log base 10 of the average count for each gene using the six different analysis strategies applied to randomly labeled samples. In each case the *P*-values should be uniformly distributed between zero and one. **(a) **Poisson model, 75th percentile normalization. **(b) **Poisson model, 75th percentile included as term. **(c) **Gaussian model, 75th percentile normalization. **(d) **Gaussian model, 75th percentile included as term. **(e)** Permutation model, 75th percentile normalization. **(f) **Permutation model, 75th percentile included as term.

Methods for RNA-Seq differential expression frequently assume that the count distribution follows a Poisson model, with a normalization factor included as an offset in the model, and this has been shown to be appropriate when technical (especially lane-to-lane) replication is considered [4,25,26]. The randomized experiment considered here includes biological replication, and it is of considerable interest to assess how well the standard Poisson model can be used to describe and assess differential expression in this circumstance. We found that the standard Poisson model is a poor fit, in the sense that *P*-values produced by this model suggest a large differential expression signal between the two randomized groups (Figures 2a, b). At a 5% level we found 5,410 differentially expressed genes where we would expect 747 (5% of 14,934). This signal is present across the entire range of expression, perhaps except for very lowly expressed genes (Figures 3a, b).

The Gaussian model using 75th percentile normalization overestimates significance as well, but the bias is much smaller than the bias from the Poisson model and is confined to genes with low counts (Figures 2c, c). When the 75th percentile is included as a regression term in the model (see Materials and methods), this bias is reduced (Figure 2d). Including the normalization constant as a term in the model reduces the effect of the normalization constant on genes with a very small number of observed counts (Figure 3d). The permutation approach shows a similar pattern of differential expression signal to the Gaussian model (Figure 2e, f), which is not surprising in light of the relatively large (n = 69) sample size. However, in this case, the bias is mostly concentrated in high-count genes (Figure 3e) as has been previously reported [27]. This bias is substantially reduced, again by including the normalization constant as a term; however, some slight read length bias is still apparent (Figure 3f), as previously described [27].

These results suggest that the commonly assumed Poisson model is not sufficiently flexible to model the variation in RNA-Seq differential expression analysis. This might be caused by the link between gene expression and variation of the gene expression assumed by the Poisson model. Methods that estimate the variance when calculating significance - such as the Gaussian model or *t*-tests - may reduce bias in differential expression analyses. When the sample size of these experiments is not sufficient to use a distributional assumption to generate *P*-values, it may be more appropriate to use a permutation procedure like we have proposed for Myrna, or to borrow strength across genes to estimate variances [28-30].

We are surprised at the substantial improvement we obtain by including the normalization factor in the model. This is equivalent to using a gene-specific correction for the sequencing effort, or in other words, genes are differentially affected by changes in sequencing depth.

These results show that more work needs to be done regarding assessing differential expression for RNA-Seq experiments, for biological replicates. The often-used Poisson distribution will vastly overestimate the amount of differential expression. Note that procedures for correcting for multiple testing, such as the Benjamini-Horchberg procedure for controlling the false discovery rate, will not affect this result as they assume that the raw *P*-values are uniformly distributed in the case of no differential expression.

### Cloud computing performance

We demonstrate Myrna's performance and scalability using the HapMap RNA-Seq dataset described in the previous section [22]. Recall this dataset consists of 1.1 billion 35-bp unpaired reads (after truncation), sequenced on the Illumina Genome Analyzer II instrument. Of the reads, 594 million (54%) align uniquely, whereas 412 million (38%) align non-uniquely and are discarded, and 97 million (8.8%) fail to align. Of the 594 million unique alignments, 189 million (32% of the reads that aligned uniquely, 17.1% of the input reads) overlap a minimal interval. Note that if gene intervals are not required to be constitutive, the number of uniquely aligned reads overlapping genes increases to 482 million (81% of the reads that aligned uniquely, 43.7% of the input reads); thus, the additional requirement that alignments overlap constitutive portions of genes reduces the usable evidence by a factor of about 2.5.

We ran the entire Myrna pipeline on this dataset using Amazon Elastic MapReduce clusters of 10, 20 and 40 worker nodes (80, 160, and 320 cores). In each case, the Myrna pipeline was executed end-to-end using scripts distributed with the Myrna package. The nodes used were EC2 Extra Large High CPU Instances, that is, virtualized 64-bit computers with 7 GB of memory and the equivalent of 8 processor cores clocked at approximately 2.5 to 2.8 Ghz. At the time of this writing, the cost of such nodes was $0.68 ($0.76 in Europe and parts of the US) per node per hour, with an Elastic MapReduce surcharge of $0.12 per node per hour.

Before running Myrna, the input read data must be stored on a filesystem accessible to the cluster. Users will typically upload and preprocess the input data to Amazon's Simple Storage Service (S3) [31] before running the rest of the Myrna pipeline. An efficient method to move data into S3 is to first allocate an Elastic MapReduce cluster of many nodes and have each node transfer a subset of the data from the source to S3 in parallel. The first stage of the Myrna pipeline performs such a bulk copy while also preprocessing the reads into the form required by later stages of the Myrna pipeline. This software was used to copy 43 gigabytes of compressed short read data from a public HTTP server located at the University of Chicago [32] to an S3 repository located in the US in about 1 hour 15 minutes (approximately 82 Mb/s effective transfer rate). The transfer cost approximately $11: about $6.40 ($7.20 in Europe and parts of the US) in cluster rental fees and about $4.30 in data transfer fees.

Transfer time depends heavily on both the size of the data and the speed of the Internet uplink at the source. Public archives like National Center for Biotechnology Information (NCBI) and the European Bioinformatics Institute (EBI) as well as many universities have very high bandwidth uplinks to Internet backbones, making it efficient to copy data between those institutions and S3. However, depending on the uplink speed at the point of origin of the sequencing data, it may be more desirable to run Myrna in either Hadoop mode or Singleton mode (see Materials and methods) on a computer or cluster located on the same local network with the sequencing instruments.

**Figure 4:**
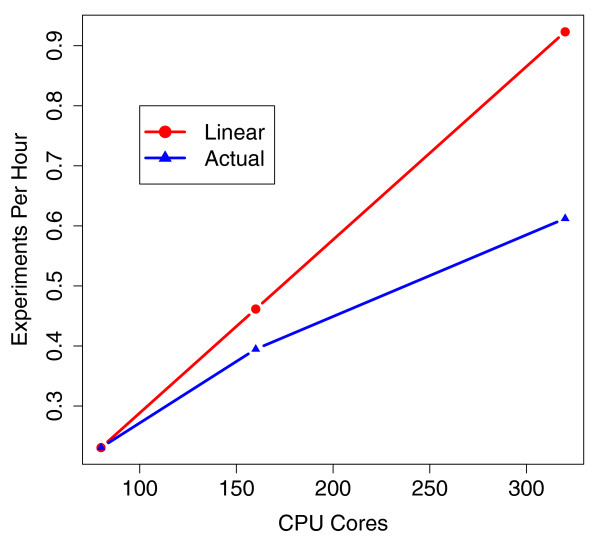
**Scalability of Myrna**. Number of worker CPU cores allocated from EC2 versus throughput measured in experiments per hour: that is, the reciprocal of the wall clock time required to conduct a whole-human experiment on the 1.1 billion read Pickrell *et al*. dataset [32]. The line labeled 'linear speedup' traces hypothetical linear speedup relative to the throughput for 80 processor cores.

To measure scalability, separate experiments were performed using 10, 20 and 40 EC2 Extra Large High CPU worker nodes (plus one master node). Table 1 presents the wall clock running time and approximate cost for each experiment. The experiment was performed once for each cluster size. The results show that Myrna is capable of calculating differential expression from 1.1 billion RNA-Seq reads in less than 2 hours of wall clock time for about $66 ($74 in Europe and parts of the US). Figure 4 illustrates scalability as a function of the number of processor cores allocated. Units on the vertical axis are the reciprocal of the wall clock time. Whereas wall clock time measures elapsed hours per experiment, its reciprocal measures experiments per hour. The straight line extending from the 80-core point represents hypothetical linear speedup, extrapolated assuming that doubling the number of processors also doubles throughput. In practice, parallel algorithms usually exhibit worse-than-linear speedup because portions of the computation are not fully parallel. For Myrna, deviation from linear speedup is primarily due to load imbalance among processors in the Align stage, but also due to a deficit of parallelism in some downstream stages (for example, Normalize and Postprocess).

**Table 1:** Myrna runtime, cost for 1.1 billion reads from the Pickrell *et al*. study [32]

EC2 nodes	1 master, 10 workers	1 master, 20 workers	1 master, 40 workers
Worker processor cores	80	160	320
Wall clock time	4h:20m	2h:32m	1h:38m
Cluster setup	4m	4m	3m
Align	2h:56m	1h:31m	54m
Overlap	52m	31m	16m
Normalize	6m	7m	6m
Statistics	9m	6m	6m
Summarize and Postprocess	13m	14m	13m
Approximate cost (location dependant)	$44.00/$49.50	$50.40/$56.70	$65.60/$73.80

Timing and cost for a Myrna experiment with 1.1 billion 35-bp unpaired reads from the Pickrell *et al*. study [32] as input. Costs are approximate and based on the pricing as of this writing, that is, $0.68 per High-CPU Extra Large instance per hour in the Northern Virginia zone and $0.78 elsewhere, plus a $0.12 per-node-per-hour surcharge for Elastic MapReduce. The table does not include the transfer of the read data from University of Chicago servers to S3, which takes a wall clock time of 1h:15 m and costs about $12. Other charges may apply and times may vary subject to, for example, congestion and Internet traffic conditions.

## Materials and methods

### Myrna computational design

Myrna is designed to run in one of three modes: 'Cloud mode' using Amazon Elastic MapReduce; 'Hadoop mode' using a Hadoop cluster; or 'Singleton mode' using a single computer. Cloud mode requires that the user have appropriate accounts and credentials set up beforehand. Cloud mode does not require any special software installation; the appropriate software is either pre-installed or automatically installed on the EC2 instances before Myrna is run. Hadoop mode requires a functioning Hadoop cluster, with Bowtie, R and Bioconductor installed on all nodes. Singleton mode requires Bowtie, R and Bioconductor to be installed on the computer, but does not require Hadoop. Singleton mode is also parallelized and can exploit a user-specified number of processors.

Myrna is designed with the Apache Hadoop [33] open source implementation of the MapReduce [34] programming model in mind. The pipeline is expressed as a series of map and reduce stages operating on 'tuples' of data. A tuple is a key/value pair, roughly analogous to a row in a database table. A map stage takes a stream of input tuples, performs a computation and outputs a stream of tuples. A reduce stage takes a stream of bundles of 'alike' tuples, where tuples are alike if their primary keys are equal. The reduce stage then performs a computation and outputs a stream of tuples. Between the map and reduce phases, the infrastructure (Hadoop in the case of the Cloud or Hadoop modes, Myrna in the case of Singleton mode) automatically executes a sort/shuffle phase that bins and sorts tuples according to primary and secondary keys, respectively, and passes the sorted bins on to the reducers. Map and reduce stages must be simple and self-contained. They cannot communicate extensively or make heavy use of global data structures. This leaves Hadoop/Myrna with significant freedom in how it distributes parallel tasks across cluster nodes and/or processors.

### Myrna workflow

#### Preprocess

Myrna's workflow is depicted in Figure 1. Each stage exploits a different type of parallelism with the aim of maximizing scalability. The first stage ('Preprocess') preprocesses a list of FASTQ files containing the input reads and installs the result on a filesystem visible to the cluster. Reads are also annotated with metadata, including the read's user-assigned sample name and the name of the file where it originated. This stage is parallel across input files, that is, files are downloaded and preprocessed simultaneously in parallel where possible.

#### Align

The second stage ('Align'; Figure 1a) aligns reads to a reference genome using Bowtie [24]. Bowtie employs a compact index of the reference sequence, requiring about 3 gigabytes of memory for the human genome. Each computer in the cluster independently obtains the index from a local or shared filesystem. When running on EC2, the index obtained here will typically be one of the pre-built indexes available publicly in S3. The user may specify options to be passed to Bowtie in this stage; the default is '-m 1', which discards alignments for reads that align multiple places. The alignment stage is parallel across reads; that is, reads are aligned simultaneously in parallel where possible.

#### Overlap

The third stage ('Overlap'; Figure 1b) calculates overlaps between alignments from the Align stage and a pre-defined collection of gene interval sets. In each instance where the 3'-most base of an alignment overlaps any base of a gene interval set, an overlap record associating the (labeled) alignment with the gene is output. By default, Myrna defines a gene interval set as the minimal set of intervals such that all contained bases are covered by all transcripts annotated for the gene. Intervals where two or more genes overlap are omitted from all gene interval sets. This is equivalent to the 'union intersection' model proposed previously [4]. Myrna allows the user to specify other models, such as the 'union' model whereby the interval set consists of the minimal set of intervals such that all contained bases are included in any exon annotation for the gene. Also, Myrna allows the user to specify which portion of the alignment to consider when overlapping with the gene interval set; for instance, instead of the 3'-most base the user can specify that the 5'-most five bases be used. The Overlap stage is parallel across alignments; that is, overlaps for distinct alignments are calculated simultaneously and in parallel where possible.

#### Normalize

The fourth stage ('Normalize'; Figure 1c) constructs a sorted vector of per-gene overlap counts for each label. A normalization factor is then calculated for each label - typically a quantile of the sample-specific gene count distribution. By default, Myrna sets the factor to the 75th percentile of the distribution of non-zero gene counts, as suggested previously [4]. Alternatively, the user may specify that Myrna use a different quantile or value, such as the median or total, as the normalization factor. The Normalize stage is parallel across labels.

#### Statistical analysis

The fifth stage ('Statistics'; Figure 1d) examines counts for each gene and calculates and outputs a *P*-value describing the probability that differences in counts observed between groups are due to chance. The Align and Overlap stages already calculated a count, *c*_*ij *_representing the number of times a read from sample *j *overlapped gene *i*. The differential expression test relates the counts to an outcome *y*_*j *_for the *j*th sample. The Normalization stage already calculated the 75th percentile, *q*_*j*_^*75*^, or another suitable summary of the count distribution for each sample.

The basic approach to differential expression is to fit a generalized linear model relating the counts *c*_*ij *_to the outcome *y*_*j*_:

$$g(E[f(c_{ij})|y_{j}])=b_{i0}+\eta_{i}\log(q)+\sum_{k=1}^{K}b_{ik}s_{k}(y_{j})$$

where *g(·) *specifies a link function (identity for Normal models, log for Poisson models) and *f(·) *is a transformation of the raw count data (identity for Poisson models, log for Normal models). The functions *s*_*k*_*(·) *can be used to specify: (1) a continuous relationship between the counts and the outcome, by setting *K *= 1 and *s*_*k*_*(·) *to be the identify function; or (2) a factor model by setting *K *= *# of groups *and *s*_*k*_*(·) *= 1(*y*_*j *_= *k*). Myrna allows the user to specify either the Gaussian or Poisson family of distributions for the generalized linear model. The normalization term, log(*q*), can be included as an offset [4], in which case *η*_*i *_= 1 for all *i*. The default setting of Myrna is to use the 75th percentile of the count distribution for each sample as the normalization factor so *q *= *q*_*j*_^*75*^.

Myrna tests the hypotheses:

$$H_{0i:}:b_{i1}=\ldots =b_{iK}=0\text{ versus }H_{1i}:b_{ik}\neq 0\;for\;some\;k$$

The hypothesis test can be performed using an asymptotic likelihood ratio test, or a permutation procedure. The permutation test is performed by first calculating the likelihood ratio statistic, *D*_*i*_, for testing *H*_*0i *_versus *H*_*1i *_for each gene. The outcome *y*_*j *_is randomly permuted *B *times; for each permutation the same procedure is applied to calculate null statistics *D*_*i*_^0b^, *b *= 1,...,*B *and *i *= 1,...,*m *where *m *is the total number of genes. Alternative statistics, like the trimmed mean statistic [9], can be implemented to try to address well known issues in RNA-Seq analysis, such as transcript length bias [27].

The Statistics stage is parallel across genes; that is, differential-expression *P*-values (both observed and null) for distinct genes are calculated simultaneously and in parallel where possible.

#### Summarize

The sixth stage ('Summarize') examines a sorted list of all *P*-values generated in the Statistics stage and compiles a list of the top N genes ranked by false discovery rate, where the parameter N is set by the user. In addition to the global significance results, more detailed statistical results and figures (see Postprocessing) are returned for the top N genes.

If a permutation test is used, the Summarize stage additionally calculates the permutation *P*-values. Permutation *P*-values are calculated as follows:

$$p_{i}=\frac{\{\#D_{j}^{0b}>D_{i};b=1,\ldots ,B\&j=1,\ldots ,m\}+1}{m•B+1}$$

This is accomplished over the course of a single linear scan of the list of observed and null statistics, sorted by statistic. The parallel infrastructure (either Hadoop or Myrna) takes care of the sorting.

Though there is a modest amount of exploitable parallelism inherent in this task, Myrna performs the Summarize stage serially (on a single processor). The lack of parallelism is mitigated by the fact that there are typically only on the order of tens of thousands or hundreds of thousands of observed and null *P*-values to examine in this stage.

#### Postprocess

The seventh stage ('Postprocess') first discards all overlap records not belonging to any top genes, which it does in parallel across all overlaps. Next, Myrna calculates per-gene *Q*-values, a false discovery rate analog of *P*-values [35]. The user specifies N whereby the N genes with the smallest *P*-values are considered 'top' genes. Finally, Myrna outputs a series of output files, including: (a) files listing all overlaps for each top gene, including alignment information that might indicate the presence of sequence variants, such as single-nucleotide polymorphisms; (b) a table with estimated RPKM values for each gene in the annotation; (c) a sorted table of all *P*-values for all genes, along with a histogram plot; (d) a sorted table of all q-values for all genes; and (e) a series of plots showing the coverage for each of the top N genes, broken down by replicate and by group. These results are then compressed and stored in the user-specified output directory.

Some stages of the Myrna pipeline may be run separately. For instance, a user may wish to preprocess a set of input reads once, then re-analyze them several times, in which case the Preprocess phase need be run only once, and the Align through Post-process stages can be re-run for subsequent analyses.

## Discussion

Myrna is a computational pipeline for RNA-Seq differential expression analysis using cloud computing. We used Myrna to analyze a large publicly available RNA-Seq dataset with over 1 billion reads. The efficiency of our pipeline allowed us to test a number of different models rapidly on even this large data set. We showed that under random labeling, a Gaussian or permutation-based testing strategy, including a normalization constant as a term in the model showed the least bias, and that the often used Poisson model vastly overestimates the amount of differential expression when biological variation is assessed. We have implemented both Gaussian and parallelized permutation tests for differential expression in Myrna.

The Myrna pipeline is complementary to existing approaches for RNA-Seq analysis - like ERANGE and Cufflinks. ERANGE attempts to recover junction reads based on the uniquely aligned reads, but only reports RPKM and does not calculate a measure of statistical significance [36]. Cufflinks is more ambitious in its attempt to fully assemble the transcriptome, but bases its differential expression statistics on the Poisson model, which we have shown may not be appropriate for biological replicates [8]. Myrna focuses on the somewhat simpler problem of differential expression analysis between genes, but uses more sophisticated statistical models and integrates the analysis in a computationally efficient pipeline.

The version of Myrna described here does not make any special attempt to align reads across exon junctions, but this is important future work. Expression signal may be lost by failing to align junction reads; Myrna's focus on just the constitutive portions of genes avoids between-sample or between-gene biases due to this policy. Users can trade off between loss of signal due to junction reads and loss of signal due to repetitive reads by adjusting the -truncate-reads option, which trims all input reads down to a given fixed length before passing them on to the alignment step. We expect that future support for counting junction reads will not severely impact Myrna's performance characteristics; its chief impact will be to add computation to the Align stage, which is currently both the biggest bottleneck and the most easily parallelizable step.

Myrna exploits the availability of multiple computers and processors where possible and can be run on the cloud using Amazon Elastic MapReduce, on any Hadoop cluster, or on a single computer (bypassing Hadoop entirely). While cloud mode allows Myrna users to tap into the vast economies of scale afforded by cloud providers, users may nonetheless prefer to run in Hadoop or Singleton mode. This may be because: cloud data transfers are inconvenient and sometimes too slow; Singleton mode is easier to use and debug when things go wrong; large, free, local Hadoop resources may be a better alternative; or privacy concerns (for example, internal review board requirements) may disallow use of the cloud. Users considering the appropriateness of the cloud for their work can also consult recent reviews and commentaries on this topic [13,14,16].

Myrna is freely available, open source software that can be downloaded from our website [37]. The RNA-Seq data used in this analysis are available from eQTL resources at the Pritchard lab [32].

## Abbreviations

BP: base pair; CPU: central processing unit; EC2: Elastic Compute Cloud; RPKM: reads per kilobase of exon model per million mapped reads; S3: Simple Storage Service.

## Authors' contributions

BL and JL designed and developed the software and collected results. BL, KH, and JL contributed to discussions on algorithms and wrote the manuscript.
